# Obesity and Risk of Colorectal Cancer: A Systematic Review of Prospective Studies

**DOI:** 10.1371/journal.pone.0053916

**Published:** 2013-01-17

**Authors:** Yanlei Ma, Yongzhi Yang, Feng Wang, Peng Zhang, Chenzhang Shi, Yang Zou, Huanlong Qin

**Affiliations:** 1 Department of Surgery, Shanghai Tenth People's Hospital, Affiliated to Tongji University, Shanghai, People’s Republic of China; 2 Department of Surgery, The Sixth People’s Hospital Affiliated to Shanghai Jiao Tong University, Shanghai, People’s Republic of China; The University of Texas M. D. Anderson Cancer Center, United States of America

## Abstract

**Background:**

Mounting evidence indicates that obesity may be associated with the risk of colorectal cancer (CRC). To conduct a systematic review of prospective studies assessing the association of obesity with the risk of CRC using meta-analysis.

**Methodology/Principal Findings:**

Relevant studies were identified by a search of MEDLINE and EMBASE databases before January 2012, with no restrictions. We also reviewed reference lists from retrieved articles. We included prospective studies that reported relative risk (RR) estimates with 95% confidence intervals (CIs) for the association between general obesity [measured using body mass index (BMI)] or central obesity [measured using waist circumference (WC)] and the risk of colorectal, colon, or rectal cancer. Approximately 9, 000, 000 participants from several countries were included in this analysis. 41 studies on general obesity and 13 studies on central obesity were included in the meta-analysis. The pooled RRs of CRC for the obese vs. normal category of BMI were 1.334 (95% CI, 1.253–1.420), and the highest vs. lowest category of WC were 1.455 (95% CI, 1.327–1.596). There was heterogeneity among studies of BMI (P<0.001) but not among studies of WC (P = 0.323).

**Conclusions:**

Both of general and central obesity were positively associated with the risk of CRC in this meta-analysis.

## Introduction

Colorectal cancer (CRC) is one of the most common cancer in the Western World [1]. Recent decades have witnessed a rapid increase in CRC morbidity in rapidly developing countries like China, especially in major cities where significant lifestyle alterations have occurred [2]. Several studies have demonstrated that lifestyle factors, such as smoking, obesity, physical inactivity, or a high-fat/low-fiber diet, might contribute to the aetiology of CRC [3], [4], [5]. The prevalence of overweight and obesity is increasing dramatically in most parts of the world, which are important for CRC prevention [6].

The relationship between obesity and the risk of CRC has been assessed by a large number of studies and review papers [7], [8], [9], [10], [11], [12], [13], [14], [15], [16], [17], [18], [19], [20], [21], [22], [23], [24], [25], [26], [27], [28], [29], [30], [31], [32], [33], [34], [35], [36], [37], [38], [39], [40], [41], [42], [43], [44], [45], [46], [47], [48], [49], [50], [51]. However, the magnitude of the association has varied widely across studies and the findings have been inconsistent. Moreover, no overall quantitative estimate has previously been reported due to different sociodemographic characteristics of participants or study methodologies in each individual study. Meanwhile, the effects of these contributing factors on the heterogeneity of the magnitude of the association were not clear and have not been systematically analyzed.

The aim of this review was to evaluate the evidence from prospective studies on general obesity [measured using body mass index (BMI)] or central obesity [measured using waist circumference (WC)] and the risk of CRC by summarizing it quantitatively with a meta-analysis approach.

## Methods

### Search Strategy

The literature search was conducted prior to January 2012 in the MEDLINE and EMBASE databases, including articles that were ahead of publication. Only studies published in English were included. The following keywords and/or Medical Subjecgt Heading (MeSH) terms were used in searching: [body mass index, BMI, obesity, overweight, or waist circumference (WC)], and (colorectal cancer or colon cancer or rectal cancer). This systematic review was planned, conducted, and reported in adherence to the standards of quality for reporting meta-analysis [52].

### Eligibility Criteria

Citations selected from this initial search were subsequently screened for eligibility. Studies were included in the meta-analysis if they met the following criteria: (1) prospective design; (2) the study of interest was the measurement of body mass index (BMI) or the waist circumference (WC) for participants; (3) the outcome of interest was colorectal, colon, or rectal cancer; and (4) the relative risk (RR) estimates with 95% confidence intervals (CIs) (or data to calculate these) were reported. Where data sets were overlapping or duplicated, only the most recent information was included. All identified studies were reviewed independently for eligibility by two authors.

### Data Extraction

Data were extracted independently by two authors and cross-checked to reach a consensus. The following variables were recorded: the first author’s last name, publication year, country where the study was performed, study period, participant sex and age, sample size, measured anthropometry and range of BMI or WC, variables adjusted for in the analysis, and RR estimates with corresponding 95% CIs for the obese vs. normal categories of BMI or the the highest vs. lowest categories of WC for participants. The study quality was assessed using the 9-star Newcastle-Ottawa Scale [53].

### Statistical Analysis

Study-specific RR estimates were combined using a random-effects model, which considers both within-study and between-study variation. Statistical heterogeneity among studies was evaluated with the Q and I^2^ statistics [54]. Sensitivity analysis was performed to evaluate the stability of the results. Each study involved in the meta-analysis was deleted each time to reflect the influence of the individual data-set to the pooled RRs.

An estimation of potential publication bias was executed by the funnel plot. Funnel plot asymmetry was assessed by the method of Egger’s linear regression test, a linear regression approach to measure funnel plot asymmetry on the natural logarithm scale of the RR [55]. Publication bias was also evaluated graphically using Begg’s funnel plot. All statistical tests were performed with the STATA software, version 11.0 (Stata Corporation, College Station, Texas). P<0.05 was considered statistically significant.

## Results

### Literature Search

**Figure 1:**
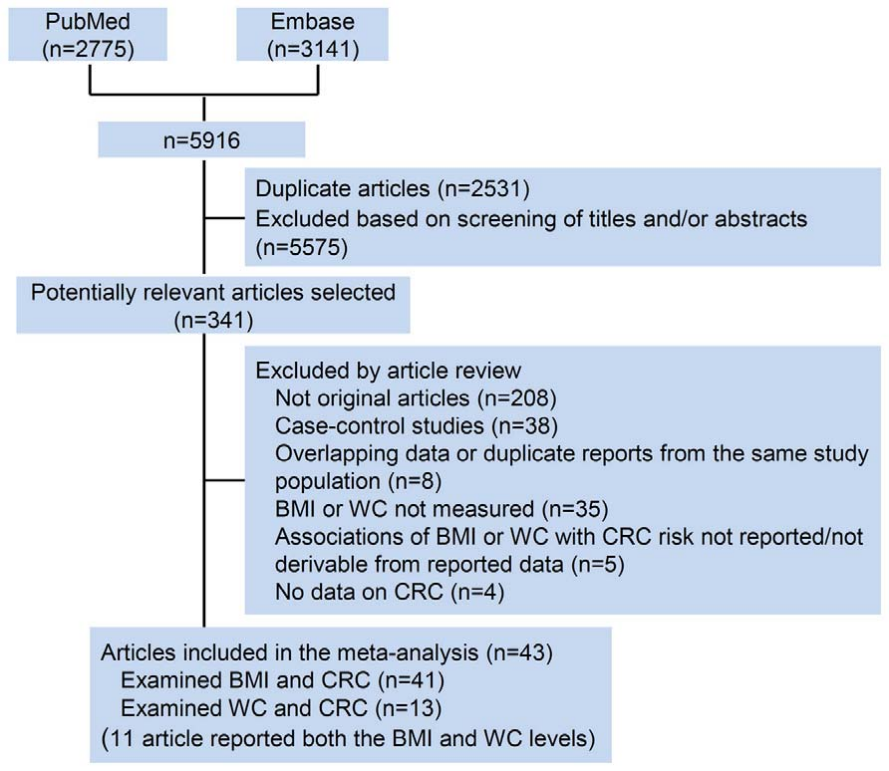
Flow diagram of the literature search process. CRC, colorectal cancer; BMI, body mass index; WC, waist circumference.

A flow diagram of our literature search is shown in **Figure 1**. Total searches yielded 5916 entries. Following the removal of 2531 duplicates, 3385 titles and abstracts were assessed and 341 articles appeared to be potentially relevant for inclusion in the review. 298 articles were excluded for the following reasons: no original articles besides editorials, comments, reviews or meta-analysis (n = 208); BMI or WC not measured (n = 35); duplicate reports from the same study population (n = 8); no data on CRC (n = 4); or associations of BMI or WC with CRC risk not reported/not derivable from reported data (n = 5). The remaining articles, including 41 on BMI [7], [8], [9], [10], [11], [12], [13], [14], [15], [16], [17], [18], [19], [20], [21], [22], [23], [24], [25], [26], [27], [28], [29], [30], [31], [32], [33], [34], [35], [36], [37], [38], [39], [40], [41], [42], [43], [44], [45], [46], [47] and 13 on WC [7], [12], [17], [22], [23], [24], [26], [28], [35], [36], [42], [45], [47] (11 article reported both the BMI and WC [7], [12], [17], [22], [23], [24], [26], [28], [35], [36], [42]), were included in the meta-analysis.

### Study Characteristics

The 41 studies on BMI measurement were published between 1992 and 2012 (**Table 1**) and involved a total of 85935 cases and 8115689 participants. Of these 41 studies, 17 were conducted in the United States, 12 in Europe, 7 in Asia, 4 in Australia and 1 in Canada. The 13 studies on WC measurement were published between 1995 and 2012 (**Table 2**) and comprised a total of 6546 cases and 817449 participants. Of those 13 studies, 7 were conducted in the United States, 3 were conducted in Europe, and 3 in Australia. Most studies provided risk estimates that were adjusted for age (36 studies), smoking (32 studies), physical activity (23 studies), alcohol consumption (23 studies). Fewer studies were adjusted for energy intake (9 studies), NSAID/aspirin use (8 studies), folate (7 studies), calcium (6 studies), diabetes (6 studies). Very few studies adjusted for CRC screening.

**Table 1:** Characteristics of prospective studies on the association between general obesity [measured using body mass index (BMI)] and risk of colorectal cancer.

Source	Location/Period	Sex	Range of age	No. of Cases(Cancer Type)	No. of Participants	Measure/Range of BMI (kg/m^2^)	RR (95% CI)	Per N-unitIncrease, RR (95% CI)	Study Quality^a^	Adjustment for Covariates
Park et al, 2011	United Kingdom1993–1997	F/M	40–79	197(CRC) (F)160 (CRC) (M)238 (CC)113 (RC)	24,244	<22.7 (Q1) (CRC) (F)≥29.4 (Q5) (CRC) (F)<23.9 (Q1) (CRC) (M)≥28.9 (Q5) (CRC) (M)	1.57 (0.91–2.73) (CRC) (F)1.06 (0.67–1.69) (CRC) (M)	Per 4-unit,0.84 (0.58–1.19) (CRC) (F)0.86 (0.60–1.24) (CRC) (M)	8	Age, sex, smoking, alcohol consumption, education, physical activity, family history of CRC, energy intake, folate, fibre, red meat intake
Odegaard et al, 2011	Singapore1993–1998	F/M	45–74	980 (CRC)596 (CC)384 (RC)	51,251	21.5–24.4 (Q3) (CRC)≥27.5 (Q5) (CRC)	1.25 (1.01–1.55) (CRC)1.48 (1.13–1.92) (CC)0.93 (0.64–1.36) (RC)	NR	8	Age, sex, smoking, alcohol consumption, year of enrollment, dialect, education, diabetes status, familial history of cancer, dietary pattern score, physical activity, sleep and energy intake
Matsuo et al, 2011	Japanaverage follow-up: 11.0 years	F/M	NR	1,924 (CRC) (F)1,534 (CC) (F)710 (PCC) (F)609 (DCC) (F)735 (RC) (F)3,055 (CRC) (M)1,919 (CC) (M)710 (PCC) (M)946 (DCC) (M)1,111 (RC) (M)	341,384	21–23 (Q3) (CRC)≥30.00 (Q7) (CRC)	1.30 (1.00–1.68) (CRC) (F)1.39 (1.02–1.90) (CC) (F)1.26 (0.79–1.99) (PCC) (F)1.76 (1.06–2.91) (DCC) (F)1.33 (0.82–2.15) (RC) (F)1.50 (1.15–1.96) (CRC) (M)1.37 (0.96–1.98) (CC) (M)1.55 (0.83–2.88) (PCC) (M)1.80 (1.11–2.92) (DCC) (M)1.85 (1.23–2.78) (RC) (M)	Per 1-unit,1.02 (1.00–1.03) (CRC) (F)1.04 (1.03–1.06) (CC) (F)1.03 (1.01–1.06) (PCC) (F)1.03 (1.00–1.06) (DCC) (F)1.00 (0.99–1.00) (RC) (F)1.03 (1.02–1.04) (CRC) (M)1.04 (1.02–1.06) (CC) (M)1.04(1.01–1.06) (PCC) (M)1.05(1.03–1.08) (DCC) (M)1.02 (1.00–1.04) (RC) (M)	7	Age, area,smoking, alcohol consumption, energy intake, red meat intake, fiber, calcium intake, folate intake and physical activity
Levi et al, 2011	Israel1967–2005	M	16–19	445 (CC)193 (RC)	939,471	<19.01(Q1) (CRC)≥23.63(Q5) (CRC)	1.69 (1.24–2.29) (CC)0.86 (0.54–1.34) (RC)	NR	8	Age, year of birth, country of origin, residence (rural or urban), immigration status, socioeconomic status, and height
Hughes et al, 2011	Netherlands1986–2002	F/M	55–69	2,316 (CRC)1,106 (CRC) (F)459 (PCC) (F)327 (DCC) (F)205 (RC) (F)1,211 (CRC) (M)327 (PCC) (M)427 (DCC) (M)299 (RC) (M)	120,852	15.4–22.1 (Q1) (CRC) (F)27.6–41.4 (Q5) (CRC) (F)16.1–23.0 (Q1) (CRC) (M)27.1–39.6 (Q5) (CRC) (M)	0.97 (0.76–1.24) (CRC) (F)0.91 (0.65–1.28) (PCC) (F)1.04 (0.72–1.50) (DCC) (F)1.07 (0.67–1.60) (RC) (F)1.25 (0.96–1.62) (CRC) (M)1.35 (0.90–1.98) (PCC) (M)1.38 (0.95–1.98) (DCC) (M)1.01 (0.67–1.51) (RC) (M)	Per 5-unit0.98 (0.88–1.10) (CRC) (F)1.02 (0.87–1.18) (PCC) (F)0.95 (0.79–1.14) (DCC) (F)1.05 (0.83–1.31) (RC) (F)Per 5-unit1.25 (1.05–1.46) (CRC) (M)1.19 (0.92–1.54) (PCC) (M)1.42 (1.13–1.79) (DCC) (M)1.02 (0.79–1.32) (RC) (M)	7	Age, smoking, alcohol consumption, energy intake, physical activity, education, family history of CRC
Oxentenko et al, 2010	United States1986–2005	F	55–69	1,464 (CRC)	36,941	≤23.45 (Q1) (CRC)≥29.52 (Q4) (CRC)	1.29 (1.10–1.51) (CRC)	NR	8	Age, age at menopause, exogenous estrogen use, oral contraceptive use, smoking, physical activity, selfreported diabetes mellitus, energy intake, red meat intake, fruits and vegetables, calcium, folate, vitamin E and alcohol consumption
Bassett et al, 2010	Australia1990–2007	F/M	40–69	569 (CC)292 (CC) (F)277 (CC) (M)	23,438 (F)16,188 (M)	23.0–25.0 (Q2) (CC)≥30.0 (Q4) (CC)	1.00 (0.70–1.44) (CC) (F)1.51 (1.00–2.28) (CC) (M)	Per 5-unit,1.01 (0.86–1.18) (CC) (F)1.39 (1.12–1.71) (CC) (M)	8	country of birth, sex, smoking, and alcohol consumption, education, red meat intake, fruit and vegetable consumption, fat intake, energy intake,
Wang et al, 2008	United States 1997–2005	F/M	>45	953 (CRC)407 (CRC) (F)314 (CC) (F)93 (RC) (F)546 (CRC) (M)402 (CC) (M)142 (RC) (M)	95,151 (CRC) (F/M)51,083 (CRC) (F)44,068 (CRC) (M)	18.5–24.9 (Q1) (CRC)≥35.0 (Q4) (CRC)	1.62 (1.04–2.54) (CRC) (F)1.40 (0.84–2.36) (CC) (F)2.67 (1.09–6.54) (RC) (F)1.76 (1.12–2.76) (CRC) (M)1.93 (1.14–3.28) (CC) (M)1.38 (0.58–3.28) (RC) (M)	NR	8	Height, smoking, education, physical activity, alcohol consumption, NSAID use, multivitamin use, and history of colorectal endoscopy, HRT use
Thygesen et al, 2008	United States 1986–2004	M	40–75	765 (CC)	46,389	20.1–22.5 (Q2) (CC)≥35.0 (Q6) (CC)	2.29 (1.23–4.26) (CC)	NR	8	Smoking, physical activity, alcohol consumption, folate, methionine, vitamin D, calcium, energy intake, red meat intake, multivitamin use, aspirin use, endoscopic screening and family history of CRC
Wang et al, 2007	United States 1992–2003	F	NR	814 (CRC)	73,842	18.5–24.9 (T1) (CRC)>30.0 (T3) (CRC)	1.19 (0.97–1.45) (CRC)	Per 5-unit,1.08 (1.00–1.17)	7	Age, smoking, education, history of colorectal endoscopy, HRT use, NSAID use, multivitamin use, physical activity, and history of diabetes
Reeves et al, 2007	United Kingdom1996–2001	F	50–64	4,008 (CRC)	1,222,630	22.5–24.9 (Q2) (CRC)≥30.0 (Q5) (CRC)	1.01 (0.94–1.09) (CRC)	Per 10-unit,1.00 (0.92–1.08)	7	Age, smoking, geographical region, socioeconomic status, reproductive history, alcohol consumption, physical activity, time since menopause and use of HRT.
Lundqvist et al, 2007	Sweden 1969–2004	F/M	43–96(Older)	513 (CC)324 (RC)	24,821 (Older)	18.5–25.0 (Q2) (CRC)≥30.0 (Q4) (CRC)	1.3 (0.9–1.8) (CC)0.7 (0.4–1.2) (RC)	Per 1-unit,1.02 (0.99–1.05) (CC)1.00 (0.97–1.04) (RC)	7	Age, sex, country, smoking, physical activity, education and diabetes
Lundqvist et al, 2007	Sweden 1969–2004	F/M	18–47 (Younger)	204 (CC)154 (RC)	43,328 (Younger)	18.50–25.0 (Q2) (CRC)≥30.0 (Q4) (CRC)	1.1 (0.5–2.5) (CC)0.9 (0.3–2.5) (RC)	Per 1-unit,1.02(0.97–1.06) (CC)1.00(0.95–1.06) (RC)	6	Age, sex, country, smoking, physical activity, education and diabetes
Driver et al, 2007	United States1982–2004	M	40–84	485 (CRC)355 (CC)100 (RC)	21,581	<25.0 (T1) (CRC)≥30 (T3) (CRC)<25.0 (CC)>25.0 (CC)<25.0 (RC)>25.0 (RC)	1.62 (1.09–2.42) (CRC)1.38 (1.11–1.70) (CC)1.19 (0.80–1.77) (RC)	NA	8	History of diabetes, physical activity, vegetable intake, cold cereal intake, vitamin C, vitamin E, and multivitamin intake
Adams et al, 2007	United States1995–2000	F/M	50–71	1,029 (CRC) (F)769 (CC) (F)278 (RC) (F)2,314 (CRC) (M)1,676 (CC) (M)677 (RC) (M)	209,436 (F)307,708 (M)	18.5–23 (Q1) (CRC)≥40 (Q8) (CRC)18.5–23 (Q1) (CC)≥40 (Q8) (CC)18.5–23 (Q1) (RC)≥35 (Q7) (RC)	1.28 (0.88–1.85) (CRC) (F)1.49 (0.98–2.25) (CC) (F)1.44 (0.92–2.25) (RC) (F)2.05 (1.45–2.91) (CRC) (M)2.39 (1.59–3.58) (CC) (M)1.00 (0.68–1.58) (RC) (M)	NR	7	Age, alcohol consumption, smoking, supplemental calcium, red meat intake, and HRT use in women
Samanic et al, 2006	Sweden 1971–1999	M	18–67	1,795 (CC)1,362 (RC)	362,552	18.5–24.9 (Q2)>30.0 (Q4)	1.74 (1.48–2.04) (CC)1.36 (1.13–1.66) (RC)	NR	7	Age and calendar year, smoking, and relative to normal weight subjects
Pischon et al, 2006	Europe 1992–2000	F/M	25–70	1,570 (CRC)984 (CC)586 (RC)563 (CC) (F)291 (RC) (F)421 (CC) (M)295 (RC) (M)	238,546(F)129,731(M)	<25.0 (T1) (CC) (F/M)≥30.0 (T3) (CC) (F/M)<21.7 (Q1) (RC) (F)≥28.9 (Q5) (RC) (F)<23.6 (Q1) (RC) (M)≥29.4 (Q5) (RC) (M)	1.07 (0.82–1.38) (CC) (F)1.06 (0.71–1.58) (RC) (F)1.41 (1.06–1.88) (CC) (M)1.05 (0.72–1.55) (RC) (M)	Per 1-unit,1.02(1.00–1.04) (CC) (F)1.05(1.02–1.08) (CC) (M)NR (RC)	8	smoking, education, alcohol consumption, physical activity, fiber intake, and red meat intake, fish and shellfish, and fruits and vegetables
MacInnis et al, 2006	Australia 1990–2003	F	40–69	212 (CC)	24,072	<25.0 (T1) (CC)≥30.0 (T3) (CC)	1.0 (0.7–1.4) (CC)	Per 5-unit,1.04 (0.90–1.20) (CC)	7	Country of birth, education and HRT use
MacInnis et al, 2006	Australia 1990–2003	F/M	27–75	229 (RC)	24,247 (F)16,867 (M)	<25.0 (T1) (RC)≥30.0 (T3) (RC)	1.2 (0.8–1.7) (RC) (F/M)1.1 (0.7–1.9) (RC) (F)1.3 (0.8–2.4) (RC) (M)	Per 5-unit,1.03 (0.88–1.21) (RC) (F/M)0.98 (0.80–1.22) (RC) (F)1.09 (0.86–1.38) (RC) (M)	7	Age, sex, and country of birth
Lukanova et al, 2006	Sweden 1985–2003	F/M	29–61	108 (CRC) (F)76 (CC) (F)31 (RC) (F)136 (CRC) (M)73 (CC) (M)58 (RC) (M)	35,362 (F)33,424 (M)	18.5–24.9 (T1) (CRC)≥30.0 (T3) (CRC)	2.01 (1.22–3.27) (CRC) (F)2.25 (1.25–3.98 (CC) (F)1.30 (0.42–3.45) (RC) (F)1.61 (0.95–2.65) (CRC) (M)1.43 (0.62–3.02) (CC) (M)1.96 (0.96–3.86) (RC) (M)	NR	6	Age, calendar year and smoking,
Larsson et al, 2006	Sweden 1997–2005	M	45–79	464 (CRC)284 (CC)120 (PC)129 (DC)180 (RC)	45,906	<23.0 (Q1) (CRC)≥30.0 (Q5) (CRC)	1.54 (1.08–2.21) (CRC)1.60 (1.03–2.48) (CC)1.43 (0.71–2.88) (PC)1.49 (0.78–2.84) (DC)1.44 (0.79–2.61) (RC)	Per 1-unit,1.04 (1.01–1.07) (CRC)	7	Age, education, smoking, family history of CRC, history of diabetes, aspirin use, and physical activity
Bowers et al, 2006	Finland 1985–2002	M	53–62	410(CR)227 (CC)183 (RC)	28,983	<18.5 (Q1) (CRC)>30.0 (Q4) (CRC)	1.66 (1.27–2.18) (CRC)1.78 (1.25–2.55) (CC)1.51 (0.99–2.29) (RC)	NR	6	Age, smoking
Ahmed et al, 2006	United States1987–2000	F/M	45–64	194 (CRC)87 (CRC) (F)107 (CRC) (M)	14,109	<25.0 (CRC) (F/M)≥35.0 (CRC) (F/M)<23.4 (CRC) (F)≥31.3 (CRC) (F)<24.7 (CRC) (M)≥29.8 (CRC) (M)	1.54 (0.9–2.8) (CRC) (F/M)1.26 (0.6–2.6) (CRC) (F)1.52 (0.9–2.7) (CRC) (M)	NR	8	Age, sex, family history of CRC, physical activity, NSAID use, aspirin use, smoking, alcohol consumption, and HRT use
Rapp et al, 2005	Austria 1985–2001	F/M	18–93	271 (CC) (F)133 (RC) (F)260 (CC) (M)138 (RC) (M)	145,931 (F/M)78,484 (F)67,447 (M)	18.5–24.9 (Q1) (CRC)≥35.0 (Q4) (CRC)	0.88 (0.43–1.81) (CC) (F)0.96 (0.38–2.39) (RC) (F)2.48 (1.15–5.39) (CC) (M)1.66 (1.01–2.73) (RC) (M)	NR	7	Age, smoking, occupational group
Otani et al, 2005	Japan 1990–2001	F/M	40–69	986 (CRC)360 (CRC) (F) 229 (CC) (F)131 (RC) (F)626 (CRC) (M)424 (CC) (M)202 (RC) (M)	102,949 (F/M)53,791 (F)49,158 (M)	<25.0 (Q1) (CRC)≥30.0 (Q4) (CRC)	0.8 (0.4–1.5) (CRC) (F)0.5 (0.2–1.4) (CC) (F)0.5 (0.1–2.1) (PC) (F)0.6 (0.1–2.5) (DC) (F)1.3 (0.5–3.1) (RC) (F)1.5 (0.9–2.5) (CRC) (M)1.4 (0.7–2.8) (CC) (M)1.8 (0.7–5.0) (PC) (M)1.3 (0.5–3.2) (DC) (M)1.6 (0.6–3.9) (RC) (M)	NR	8	Age, Public Health Center areas, smoking, alcohol consumption, miso soup intake, refraining from salty foods and animal fats
Oh et al, 2005	Korea 1992–2001	M	≥20	953 (CC) (M)1,563 (RC) (M)	781,283	18.5–22.9 (Q2) (CRC)≥30.0 (Q6) (CRC)	1.92 (1.15–3.22) (CC)1.08 (0.56–2.10) (RC)	NR	8	Age, smoking, alcohol consumption, physical activity, family history of cancer, and residency area at baseline
Kuriyama et al, 2005	Japan/1984–1992	F/M	≥40	270 (CRC) (F/M)115 (CRC) (F)72 (CC) (F)42 (RC) (F)155 (CRC) (M)88 (CC) (M)67 (RC) (M)	27,539 (F/M)15,054 (F)12,485 (M)	18.5–24.9 (Q1) (CRC)≥30.0 (Q4) (CRC)	2.06 (1.03–4.13) (CRC) (F)2.25 (0.95–5.33) (CC) (F)1.03 (0.13–8.01) (PC) (F)2.86 (0.98–8.37) (DC) (F)1.21 (0.29–5.14) (RC) (F)1.78 (0.73–4.38) (CRC) (M)1.30 (0.32–5.37) (CC) (M)1.71 (0.23–12.92) (PC) (M)1.41 (0.19–10.52) (DC) (M)2.41 (0.74–7.85) (RC) (M)	NR	8	Age, smoking, alcohol consumption, red meat intake, consumption of fish, fruits, vegetables, bean-paste soup, type of health insurance, menopausal status, parity, age at menarche, age at end of first pregnancy
Engeland et al, 2005	Norway 1963–2001	F/M	20–74	47,117 (CRC)24,130 (CRC) (F)16,638 (CC) (F)7,492 (RC) (F)22,987 (CRC) (M)13,805 (CC) (M)9,182 (RC) (M)	1,037,077 (F)962,901 (M)	18.5–24.9 (Q2) (CRC)≥30.0 (Q4) (CRC)	1.06 (1.02–1.10) (CRC) (F)1.07 (1.02–1.12) (CC) (F)1.04 (0.97–1.11) (RC) (F)1.40 (1.32–1.48) (CRC) (M)1.49 (1.39–1.60) (CC) (M)1.27 (1.16–1.38) (RC) (M)	NR	6	Age
Wei et al, 2004	United States1980–2000	F/M	30–75	1139 (CC) (F/M)339 (RC) (F/M)671 (CC) (F)204 (RC) (F)452 (CC) (M)132 (RC) (M)	87,733 (CRC) (F)46,632 (CRC) (M)	<23.0 (Q1) (CRC)>30.0 (Q4) (CRC)	1.39 (1.14–1.69) (CC) (F/M)1.40 (0.96–2.03) (RC) (F/M)1.28 (1.10–1.62) (CC) (F)1.56 (1.01–2.42) (RC) (F)1.85 (1.26–2.72) (CC) (M)1.03 (0.49–2.14) (RC) (M)	Per 5-unit,1.06 (1.03–1.10) (CRC)	8	Age, family history of CRC, physical activity, red meat intake, alcohol consumption, calcium, folate, height, smoking, history of endoscopy and gender in combined cohort
Moore et al,2004	United States1948–1999	F/M	30–54	157 (CC) (F/M)86 (CC) (F)71 (CC) (M)	3764 (Younger)	18.5–25 (T1) (CC)≥30 (T3) (CC)	1.5 (0.92–2.5) (CC) (F/M)1.6 (0.90–3.0) (PC) (F/M)1.4 (0.55–3.6) (DC) (F/M)1.3 (0.65–2.7) (CC) (F)2.0 (0.98–4.2) (CC) (M)	NR	8	Age, sex, education, height, alcohol consumption, smoking, and physical activity
Moore et al, 2004	United States1948–1999	F/M	55–79	149 (CC) (F/M)80 (CC) (F)69 (CC) (M)	3802 (Older)	18.5–25.0 (T1) (CC)≥30.0 (T3) (CC)	2.4 (1.5–3.9) (CC) (F/M)2.9 (1.6–5.2) (PC) (F/M)1.8 (0.75–4.3) (DC) (F/M)1.9 (0.98–3.5) (CC) (F)3.7 (1.7–8.1) (CC) (M)	NR	8	Age, sex, education, height, alcohol consumption, smoking, physical activity
MacInnis et al, 2004	Australia 1991–2002	M	27–75	153 (CC)	17,049	<24.8 (Q1) (CC)>29.2 (Q4) (CC)	1.70 (1.10–2.80) (CC)	Per 5-unit,1.29 (1.04–1.60) (CC)	7	Age, country of birth, and education
Lin et al, 2004	United StatesFrom 1993, average follow-up: 8.7 years	F	≥45	158 (CC)	39,876	<23.0 (Q1) (CRC)≥30.0 (Q5) (CRC)	1.67 (1.08–2.59) (CRC)1.73 (1.05–2.85) (CC)2.59 (1.34–5.01) (PC)0.93 (0.41–2.14) (DC)1.55 (0.64–3.77) (RC)	NR	8	Age, randomized treatment assignment, family history of CRC, history of colon polyps, physical activity, smoking, baseline aspirin use, red meat intake, alcohol consumption, menopausal status and baseline postmenopausal hormone therapy use
Shimizu et al, 2003	Japan 1993–2000	F/M	≥35	134 (CRC) (F)89 (CC) (F)41 (RC) (F)161 (CRC) (M)104 (CC) (M)58 (RC) (M)	29,051 (F/M)15,659 (F)13,392 (M)	≤21.2 (T1) (CRC)≥23.6 (T3) (CRC)	1.22 (0.69–2.15) (CC) (F)0.83 (0.35–1.99) (RC) (F)2.11 (1.26–3.53) (CC) (M)0.83 (0.42–1.64) (RC) (M)	NR	7	Age, height, alcohol consumption, smoking, education, physical activity
Terry et al, 2002	Canada 1980–1993	F	40–59	527 (CRC)363 (CC)164 (RC)	89,835	<25.0(T1) (CRC)≥30 (T3) (CRC)	1.08 (0.82–1.41) (CRC)0.95 (0.67–1.34) (CC)0.81 (0.48–1.38) (PC)1.31 (0.79–2.16) (DC)1.35 (0.87–2.07) (RC)	NR	7	Age, smoking, education, physical activity, oral contraceptive use, HRT, and parity
Terry et al, 2001	Sweden 1987–1998	F	40–76	460 (CRC)291 (CC)118 (PC)101 (DC)159 (RC)	61,463	<22.0 (Q1) (CRC)>26.7 (Q4) (CRC)	1.24 (0.95–1.62) (CRC)1.21 (0.86–1.70) (CC)1.13 (0.66–1.94) (PC)1.21 (0.67–2.19) (DC)1.32 (0.83–2.08) (RC)	NR	8	Age, education, quartiles of intakes of energy, alcohol, red meat intake, total fat, folate, vitamin D, vitamin C and calcium
Kaaks et al, 2000	United States1985–1998	F	35–65	100 (CRC)73 (CC)	14,275	Q1 (CRC)Q5 (CRC)	2.83 (1.23–6.54) (CRC)3.07 (1.12–8.41) (CC)	NR	8	Age, menopausal status, day of menstrual cycle, and time of last food consumption, smoking
Schoen et al, 1999	United States 1989–1996	F/M	≥65	102 (CRC)	5,849	14.6–23.2 (Q1) (F)29.61–58.8 (Q4) (F)15.6–23.9 (Q1) (M)28.51–46.2 (Q4) (M)	1.4 (0.8–2.5) (CRC) (F/M)	Per 1-unit,1.1 (0.9–1.3) (CRC) (F/M)	6	Age, sex, and physical activity
Ford et al, 1999	United States1971–1992	F/M	25–74	222 (CC) (F/M)118 (CC) (F)104 (CC) (M)	13,420 (F/M)7,914 (F)5,506 (M)	<22.0 (CC) (Q1)≥30 (CC) (Q6)	2.79 (1.22–6.35) (CC) (F/M)2.74 (1.04–7.25) (CC) (F)2.95 (0.99–8.74) (CC) (M)	NR	8	Age, sex, race, education, smoking, serum cholesterol concentration, recreational exercise, physical activity, and alcohol consumption
Singh et al, 1998	United States1976–1982	F/M	≥25	83 (CC) (F)59 (CC) (M)	32,051	<22.5(T1) (CC)>25.6(T3) (CC)	1.33(0.88–2.06) (CC) (F/M)1.05(0.63–1.75) (CC) (F)2.63(1.12–6.13) (CC) (M)	NR	7	Age, sex, and family history of CRC
Chyou et al, 1996	United States1965–1995	M	≥45	330 (CC) (M)123 (RC) (M)	7,945	<21.7 (Q1) (CRC)≥25.8 (Q4) (CRC)	1.38 (1.01–1.90) (CC)0.63 (0.38–1.04) (RC)	Per 1-unit,1.06 (1.03–1.10) (CRC)	6	Age
Bostick et al, 1994	United States1986–1990	F	55–69	212 (CC) (F)	35,215 (F)	<22.9 (Q1) (CC)>30.6 (Q5) (CC)	1.41 (0.90–2.23) (CC)	NR	8	Age, energy intake, height, parity, total vitamin E intake, a total vitamin E by age interaction term, and vitamin A supplement intake
Lee et al, 1992	United States1962–1988	M	NA	290 (CC)	17,595	<22.5 (Q1) (CC)z26.0 (Q5) (CC)	1.01.52 (1.06–2.17) (CC)	Per 1-unit,1.08 (1.04–1.13) (CC)	7	Age, physical activity, and family history of cancer

Abbreviations: BMI, body mass index; CRC, colorectal cancer; CC, colon cancer; RC, rectal cancer; DCC, distal colon cancer; PCC, proximal colon cancer; F, female; M, male; T, tertile; Q, quartile/quintile; HRT, hormone replacement therapy; NR, not report.

a Study quality was judged based on the Newcastle-Ottawa Scale (range, 1–9 stars).

**Table 2:** Characteristics of prospective studies on the association between central obesity [measured using waist circumference (WC)] and risk of colorectal cancer.

Source	Location/Period	Sex	Age	No. of Cases(Cancer Type)	No. of Participants	Measure/Range of waist circumstance (cm)	RR (95% CI)	Per N-unitIncrease, RR (95% CI)	Study Quality^a^	Adjustment for Covariates
Park et al, 2011	United Kingdom 1993–1997	F/M	40–79	197 (CRC) (F)160 (CRC) (M)	24,244 (F/M)13078 (F)11166 (M)	<88.0 (Q1) (CRC)≥103.3 (Q5) (CRC)	1.65 (0.97–2.86) (CRC) (F)0.86 (0.55–1.36) (CRC) (M)	Per 10-unit1.41 (1.06–1.87) (CRC) (F)1.06 (0.77–1.46) (CRC) (M)	8	Age, sex, smoking, alcohol consumption, education, exercise, family history of CRC, energy intake, folate, fibre, total meat and processed meat intakes, height
Oxentenko et al, 2010	United States 1986–2005	F	55–69	1,464 (CRC)	36,941	≤77.15 (Q1) (CRC)≥96.53 (Q4) (CRC)	1.32 (1.11–1.56) (CRC)	NR	8	Age, exogenous estrogen use, oral contraceptive use, smoking, physical activity level, selfreported diabetes mellitus, total energy intake, total fat, red meat, fruits and vegetables, calcium, folate, vitamin E and alcohol consumption
Wang et al, 2008	US 1997–2005	F/M	>45	953 (CRC)407 (CRC) (F)546 (CRC) (M)	95,151 (F/M)51,083 (F)44,068 (M)	<85.0 (Q1) (CRC) (F)≥110.0 (Q4) (CRC) (F)<95.0 (Q1) (CRC) (M)≥120.0 (Q4) (CRC) (M)	1.75 (1.20–2.54) (CRC) (F)1.54 (1.00–2.37) (CC) (F)2.65 (1.23–5.71) (RC) (F)1.68 (1.12–2.53) (CRC) (M)2.05 (1.29–3.25) (CC) (M)1.02 (0.43–2.42) (RC) (M)	NR	8	Height, education, physical activity, smoking, alcohol consumption, NSAID use, multivitamin use, and history of colorectal endoscopy, HRT use
Pischon et al, 2006	Europe 1992–2000	F/M	25–70	1,570 (CRC)56 2 (CC) (F)418 (CC) (M)291 (RC) (F)293 (RC) (M)	238,546 (F)129,731 (M)	<70.2 (Q1) (CRC) (F)≥89.0 (Q5) (CRC) (F)<86.0 (Q1) (CRC) (M)≥103.0 (Q5) (CRC)(M)	1.48 (1.08–2.03) (CC) (F)1.23 (0.81–1.86) (RC) (F)1.39 (1.01–1.93) (CC) (M)1.27 (0.84–1.91) (RC) (M)	Per 5-unit,1.07(1.03–1.12) (CC) (F)1.10(1.05–1.56) (CC) (M)NR (RC)	8	Smoking, education, alcohol consumption, physical activity, red and processed meat intake, fish and shellfish, fiber, fruits and vegetables
MacInnis et al, 2006	Australia 1990–2003	F	40–69	212 (CC)	24,072	<80.0 (T1) (CC)≥88.0 (T3) (CC)	1.4 (1.0–1.9) (CC)	Per 10-unit1.14 (1.02–1.28) (CC)	7	country of birth, highest level of education and HRT use
MacInnis et al, 2006	Australia 1990–2003	F/M	27–75	229 (RC)	24,247 (F)16,867 (M)	<80.0 (T1) (RC) (F)≥88.0 (T3) (RC) (F)<94.0 (T1) (RC) (M)≥102.0 (T3) (RC) (M)	1.4 (1.0–1.9) (RC) (F/M)1.4 (0.8–2.2) (RC) (F)1.4 (0.9–2.2) (RC) (M)	Per 10-unit1.12(0.99–1.27) (RC) (F/M)1.10 (0.93–1.30) (RC) (F)1.15 (0.97–1.36) (RC) (M)	7	Age, sex, and country of birth
Larsson et al, 2006	Sweden 1997–2005	M	45–79	407 (CRC)252 (CC)110 (PC)112 (DC)180 (RC)	45,906	<88.0 (Q1) (CRC)≥104.0 (Q5) (CRC)	1.29 (0.90–1.85) (CRC)1.44 (0.93–2.24) (CC)1.66 (0.84–3.27) (PC)1.62 (0.80–3.27) (DC)1.24 (0.68–2.25) (RC)	NR	7	Age, education, family history of colorectal cancer, history of diabetes, smoking, aspirin use, and leisure-time physical activity
Ahmed et al, 2006	United States 1987–2000	F/M	45–64	194 (CRC)87 (CRC) (F)107 (CRC) (M)	14,109	<88.0 (Low) (F) (CRC)≥88.0 (High) (F)) (CRC)<102 (Low) (M) (CRC)102 (High) (M) (CRC)	1.40 (1.0–1.9) (CRC) (F/M)	NR	8	Age, gender, family history of colorectal cancer, physical activity, NSAID use, aspirin use, smoking, alcohol consumption, current HRT use
Moore et al, 2004	United States 1948–1999	F/M	30–54	157 (CC) (F/M)86 (CC) (F)71 (CC) (M)	3,764 (Younger)	<81.3 (Q1) (F)≥99.1 (Q4) (F)<83.8 (Q1) (M)≥101.6 (Q4) (M)	2.0 (1.1–3.7) (CC) (F/M)1.7 (0.82–3.7) (PC) (F/M)2.6 (0.87–7.6) (DC) (F/M)1.8 (0.78–4.3) (CC) (F)2.4 (0.99–5.7) (CC) (M)	NR	8	Sex, education, age, height, alcohol intake, cigarettes per day, and physical activity
Moore et al,2004	United States 1948–1999	F/M	55–79	149(CC) (F/M)80(CC) (F)69(CC) (M)	3,802 (Older)	<81.3 (Q1) (F)≥99.1 (Q4) (F)<83.8 (Q1) (M)≥101.6 (Q4) (M)	2.6 (1.3–5.2) (CC) (F/M)3.1 (1.3–7.9) (PC) (F/M)1.9 (0.65–5.4) (DC) (F/M)2.3 (0.86–6.3) (CC) (F)3.3 (1.3–8.8) (CC) (M)	NR	8	Sex, education, age, height, alcohol consumption, smoking, and physical activity
MacInnis et al, 2004	Australia 1991–2002	M	27–75	153 (CC) (M)	17,049	<87.0 (Q1) (CC)>99.3 (Q4) (CC)	2.1 (1.3–3.5) (CC)	Per 10-unit,1.37 (1.18–1.60) (CC)	7	Age at attendance, country of birth, and education
Schoen et al, 1999	United States1989–1996	F/M	≥65	102 (CRC)	5,849	32.5–82.0 (Q1) (F)101.2–167 (Q4) (F)69.0–91.0 (Q1) (M)104.1–145.5 (Q4) (M)	2.2 (1.2–4.1) (CRC)	NR	6	Age, sex, and physical activity
Martinez et al, 1997	United States1986–1992	F	30–55	396 (CC)159 (PC)185 (DC)	89,448 (F)	<27.5 in. (Low)>34.0 in. (High)	1.48 (0.89–2.46) (CC)	NR	8	Age, smoking, family history of CRC, physical activity, postmenopausal hormone use, aspirin use, red meat intake, and alcohol consumption
Giovannucci et al,1995	United States1987–1992	M	40–75	203 (CC)	47,723 (M)	<35.0 in. (Q1)≥43 in. (Q5)	2.56 (1.33–4.96) (CC)	NR	8	Age, smoking, history of endoscopic screening, previous polyp diagnosis, parental history of CRC, physical activity, aspirin use, intake of folate, methione, alcohol, dietary fiber, total energy, and red meat

Abbreviations: WC, waist circumference; CRC, colorectal cancer; CC, colon cancer; RC, rectal cancer; DCC, distal colon cancer; PCC, proximal colon cancer; F, female; M, male; T, tertile; Q, quartile/quintile; HRT, hormone replacement therapy; NR, not report.

a Study quality was judged based on the Newcastle-Ottawa Scale (range, 1–9 stars).

### Obese vs. Normal Category of BMI

**Figure 2:**
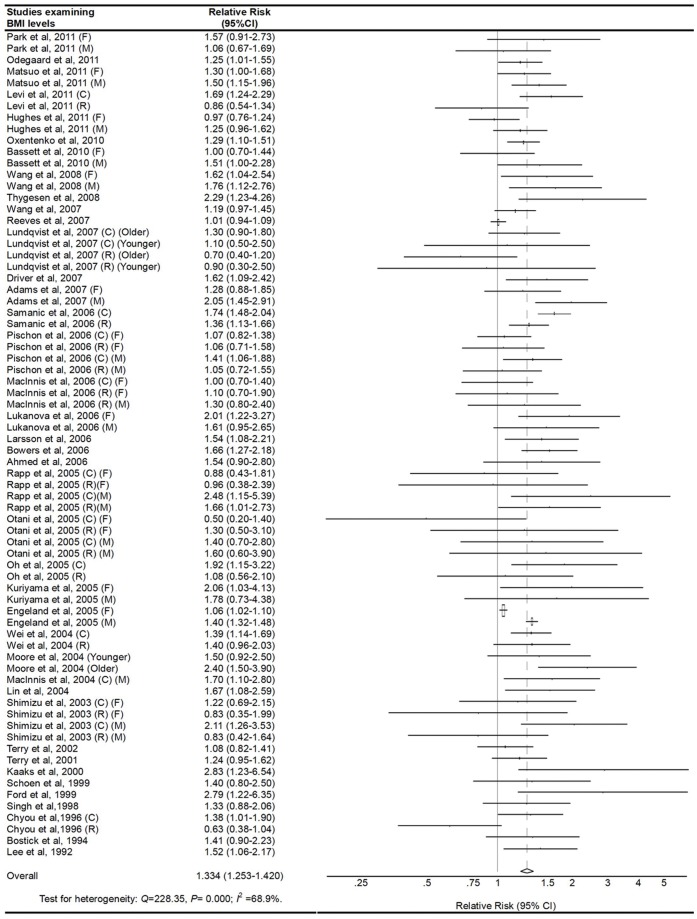
Adjusted relative risks of colorectal cancer for the obese vs. normal category of BMI. The size of each square is proportional to the weight of the study (inverse of variance). CI, confidence interval; BMI, body mass index; C, colon cancer; R, rectal cancer; F, female; M, male.

The multivariable-adjusted RRs for each study and combination of all studies for the obese vs. normal categories of BMI levels are shown in **Figure 2**. Results from the studies on BMI levels in relation to CRC risk were inconsistent. The pooled RRs of CRC for the obese vs. normal categories of BMI levels were 1.334 (95% CI, 1.253–1.420). There was high heterogeneity among studies (P<0.001, I^2^ = 68.9%), so we conducted subgroup meta-analysis and sensitivity analysis to explore the sources of heterogeneity. Through omitting one study at a time and calculating the pooled RRs for the remainder of the studies, there were no changes in the direction of the effect when any one study was excluded. For example, when we excluded the study wrote by Engeland et al. (the study that carried the most weight) from the analysis, the summarized RR remained significant (RR = 1.344, 95%CI: 1.258–1.436), and the heterogeneity was still significant (P<0.001). This analysis confirmed the stability of the positive association between BMI and the risk of CRC. The same method was also suitable for the following analysis.

### High vs. Low Category of WC

**Figure 3:**
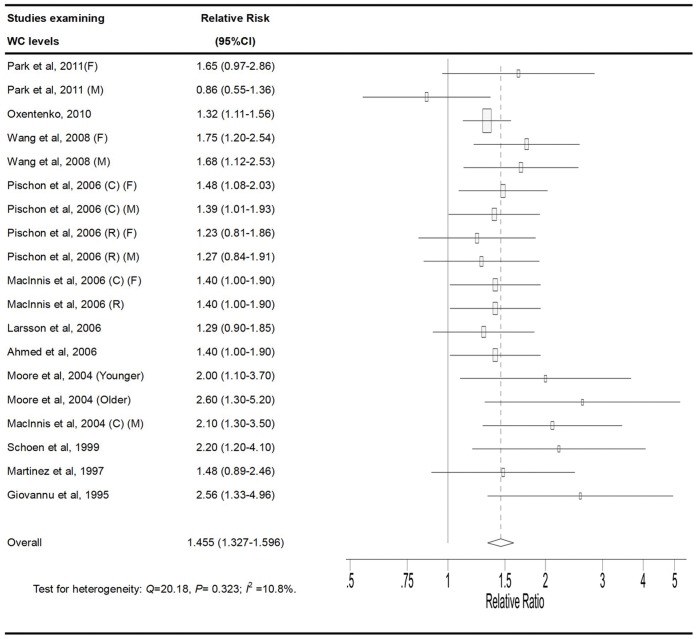
Adjusted relative risks of colorectal cancer for the highest vs. lowest categories of WC. The size of each square is proportional to the weight of the study (inverse of variance). CI: confidence interval; WC, waist circumference; C, colon cancer; R, rectal cancer; F, female; M, male.

The multivariable-adjusted RRs for each study and combination of all studies for the high vs. low categories of WC levels are shown in **Figure 3**. The pooled RRs of CRC for the high vs. low categories of WC levels were 1.455 (95% CI, 1.327–1.596). There was no statistically significant heterogeneity among the studies of WC measurement (P = 0.323, I^2^ = 10.8%).

### Stratifying Analysis

Stratifying by geographic region, the pooled RRs of CRC for the obese vs. normal categories of BMI were 1.465 (95% CI, 1.325–1.619) for studies conducted in the United States, 1.250 (95% CI, 1.149–1.360) for studies conducted in Europe, 1.351 (95% CI, 1.181–1.546) for studies conducted in Asia, and 1.203 (95% CI, 1.003–1.445) for studies conducted in Australia. The pooled RRs of CRC for the highest vs. lowest categories of WC level were 1.612 (95% CI,1.379–1.885) for studies conducted in the United States, 1.368 (95% CI, 1.215–1.541) for studies in Europe, and 1.506 (95% CI, 1.216–1.865) for studies in Australia. There was no statistically significant heterogeneity among studies of BMI (United States: P = 0.052, I^2^ = 34.8%; Asia: P = 0.165, I^2^ = 25.1%; Australia: P = 0.350, I^2^ = 10.3%) and among studies of WC levels (United States: P = 0.227, I^2^ = 24.3%; Europe: P = 0.520, I^2^ = 0%; Australia: P = 0.345, I^2^ = 6.0%), with stratification by geographic region (**Table 3**). However, there was high heterogeneity among studies from Europe (P<0.001, I^2^ = 77.5%) among studies of BMI.

**Table 3:** The association between general obesity or central obesity and the risk of colorectal cancer stratifying analysis by geographic region, anatomical subsite, and sex.

	BMI		WC	
	RR (95% CI)	Heterogeneity (P-value, I^2^)	RR (95% CI)	Heterogeneity (P-value, I^2^)
**Anatomical subsite**				
Colorectal cancer	1.334 (1.253–1.420)	<0.001, 68.9%	1.455 (1.327–1.596)	0.323, 10.8%
Colon cancer	1.470 (1.348–1.602)	<0.001, 71.3%	1.613 (1.417–1.837)	0.573, 0.0%
Proximal colon cancer	1.296 (1.109–1.514)	0.058, 40.5%	1.873 (1.118–3.136)	0.773, 0.0%
Distal colon cancer	1.367 (1.164–1.605)	0.798, 0.0%	1.942 (1.250–3.017)	0.507, 0.0%
Rectal cancer	1.149 (1.099–1.201)	0.048, 29.3%	1.349 (1.114–1.634)	0.582, 0.0%
**Geographic region**				
US	1.465 (1.325–1.619)	0.052, 34.8%	1.612 (1.379–1.885)	0.227, 24.3%
Europe	1.250 (1.149–1.360)	<0.001, 77.5%	1.368 (1.215–1.541)	0.520, 0.0%
Asia	1.351 (1.181–1.546)	0.165, 25.1%	NR	NR
Australia	1.203 (1.003–1.445)	0.350, 10.3%	1.506 (1.216–1.865)	0.345, 6.0%
**Sex**				
Colorectal cancer				
Men	1.467 (1.363–1.579)	0.043, 31.9%	1.477 (1.300–1.677)	0.135, 30.2%
Women	1.153 (1.078–1,234)	0.026, 37.2%	1.442 (1.296–1.604)	0.834, 0.0%
Colon cancer				
Men	1.547 (1.467–1.632)	0.585, 0.0%	1.812 (1.464–2.242)	0.308, 15.9%
Women	1.228 (1.097–1.374)	0.014, 46.4%	1.498 (1.253–1.791)	0.955, 0.0%
Rectal cancer				
Men	1.238 (1.112–1.378)	0.154, 25.1%	1.281 (0.990–1.657)	0.934, 0.0%
Women	1.070 (1.006–1.138)	0.727, 0.0%	1.495 (1.025–2.181)	0.224, 33.1%

**Abbreviations:** BMI, body mass index; WC, waist circumference; CI, confidence interval; HR, hazard ratio; NR, not reported.

Among the 32 studies that provided results on BMI levels in relation to colon cancer risk, the RR was 1.470 (95% CI, 1.348–1.602). 25 cohort studies on BMI levels and rectal cancer risk were identified. The RR for rectal cancer was 1.149 (95% CI, 1.099–1.201). The results showed that a higher BMI results in an equal increase in risk for colon cancer and rectal cancer. However, there was high heterogeneity among studies on BMI levels in relation to colon cancer risk (P<0.001, I^2^ = 71.3%). Among 9 studies that provided results on WC levels in relation to colon cancer risk, the RR was 1.613 (95% CI,1.417–1.837). 5 studies reported RR estimates for the highest vs. the lowest category of WC levels and risk of rectal cancer. The RR for rectal cancer was 1.349 (95% CI, 1.114–1.634). There was no statistically significant heterogeneity among studies of WC (colon cancer: P = 0.573, I^2^ = 0%; rectal cancer: P = 0.582, I^2^ = 0%) with stratification by colon and rectum (**Table 3**).

When we stratified the analysis by proximal colon and distal colon, the pooled RRs of proximal colon cancer (9 studies) and distal colon cancer (9 studies) for the obese vs. normal categories of BMI were 1.296 (95% CI, 1.109–1.514) and 1.367 (95% CI, 1.164–1.605), respectively. The results showed that there is a strong association for higher BMI levels with proximal colon cancer or distal colon cancer. There was no statistically significant heterogeneity among studies of BMI (proximal colon cancer: P = 0.058, I^2^ = 40.5%; distal colon cancer: P = 0.798, I^2^ = 0%) with stratification by proximal colon and distal colon. Meanwhile, the pooled RRs of proximal colon cancer (2 studies) and distal colon cancer (2 studies) were 1.873 (95% CI, 1.118–3.136) and 1.942 (95% CI, 1.250–3.017) for the highest vs. lowest categories of WC levels, respectively. The results showed that a higher WC levels Is associated with an increased risk for proximal colon or distal colon cancer. There was no statistically significant heterogeneity among studies of WC level (proximal colon cancer: P = 0.773, I^2^ = 0%; distal colon cancer: P = 0.507, I^2^ = 0%) with stratifying by proximal colon and distal colon (**Table 3**).

Stratifying by sex, the pooled RRs of CRC (28 studies) from male and female CRC studies for the obese vs. normal categories of BMI were 1.467 (95% CI,1.363–1.579) and 1.153 (95% CI, 1.078–1.234), respectively. The pooled RRs of CRC from male and female CRC studies for the highest vs. lowest categories of WC level were 1.477 (95% CI, 1.300–1.677) and 1.442 (95% CI, 1.296–1.604), respectively. Further stratifying by sex and colon subsites, the pooled RRs of colon cancer from male and female CRC studies for the obese vs. normal categories of BMI were 1.547 (95% CI, 1.467–1.632) and 1.228 (95% CI, 1.097–1.374), respectively. The pooled RRs of colon cancer from male and female CRC studies for the highest vs. lowest categories of WC level were 1.812 (95% CI, 1.464–2.242) and 1.498 (95% CI, 1.253–1.791), respectively. Further stratifying by sex and rectum subsites, the pooled RRs of rectal cancer from male and female CRC studies for the obese vs. normal categories of BMI were 1.238 (95% CI, 1.112–1.378) and 1.070 (95% CI, 1.006–1.138), respectively. The pooled RRs of rectal cancer from male and female CRC studies for the highest vs. lowest categories of WC level were 1.281 (95% CI, 0.990–1.657) and 1.495 (95% CI, 1.025–2.181), respectively (**Table 3**). There was no statistically significant heterogeneity among studies of WC with stratification by sex and anatomical site. However, there was high heterogeneity among studies of BMI with stratification by sex and anatomical site.

### Publication Bias

**Figure 4:**
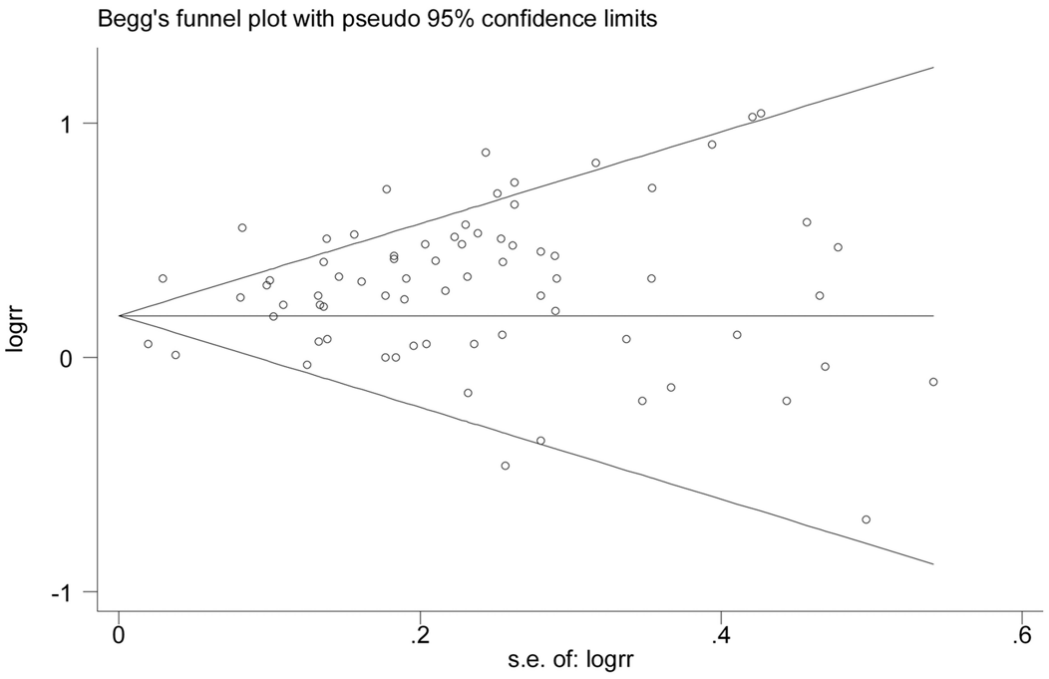
Begg’s funnel plot for Identification of publication bias in all studies for the obese vs. normal category of BMI and risk of colorectal cancer.

**Figure 5:**
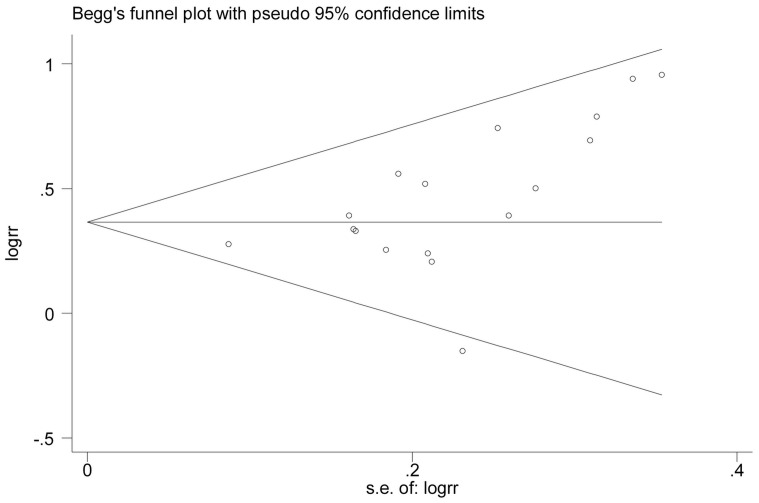
Begg’s funnel plot for Identification of publication bias in all studies for the highest vs. lowest categories of WC and risk of colorectal cancer.

The Egger’s test showed no evidence of publication bias for BMI (P = 0.166) or WC levels (P = 0.937), respectively (**Figure 4** **and** **Figure 5**).

## Discussion

The present meta-analysis summarizes the results of prospective studies, including 41 studies [7], [8], [9], [10], [11], [12], [13], [14], [15], [16], [17], [18], [19], [20], [21], [22], [23], [24], [25], [26], [27], [28], [29], [30], [31], [32], [33], [34], [35], [36], [37], [38], [39], [40], [41], [42], [43], [44], [45], [46], [47] on BMI levels with a total of 85, 935 cases and including 13 studies [7], [12], [17], [22], [23], [24], [26], [28], [35], [36], [42], [45], [47] on WC levels with a total of 6,546 cases. The results indicated that higher BMI and WC levels were positively associated with CRC risk. Analyses stratified by the anatomical site suggested that both of higher BMI and WC levels caused an equal increasing risk for colon cancer and rectal cancer. When the analysis was stratified by proximal colon and distal colon, the results showed that there was a strong risk for proximal colon cancer or distal colon cancer higher with high BMI or WC levels. Stratifying by geographic region, the results revealed that higher BMI and WC levels were positively associated with CRC risk in United States, Europe, Asia, or Australia. In additon, when the analysis was stratified by sex and anatomical site, the results showed that there was an increased risk of CRC development associated with higher BMI and WC levels for male or female.

Obesity is considered one important risk factor for many types of solid cancers, especially for CRC [56]. Previous reviews have indicated that obesity is associated with 7% to 60% greater risk of CRC compared with normal weight individuals [3], [57]. However, the mechanisms that might underlie the association between excess weight and CRC remain unclear. Currently, several possibilities have been hypothesized. Two hormonal systems – the insulin/insulin-like growth factor (IGF) axis and adipokines (adiponectin and leptin) – are the most studied candidates. First, the involvement of insulin and IGF-1 in colorectal carcinogenesis has been supported by experimental and clinical studies [58]. Circulating total IGF-I, a major determinant of free IGF-I concentrations, is associated with increased risk of colorectal advanced adenomas and cancer [59], [60]. The main reason is that increased free IGF-I with concomitant changes of environment mitogenesis and anti-apoptosis in the cellular favouring tumour formation [61]. Moreover, there is an increased risk of CRC development associated with type 2 diabetes [62]. Second, previous studies have demonstrated that the fat itself can also influence CRC risk [63]. Adipocytes and preadipocytes could promote proliferation of CRC cells [64]. Fatty acid synthase over-expression has been shown to be associated with CRC phenotype [65]. Adipokines such as adiponectin, leptin are also associated with the risk of CRC. Adiponectin as an insulin-sensitizing agent and a negative regulator of angiogenesis is secreted mainly from visceral adipose tissue, which could inhibit CRC growth in animal models, and its circulating concentrations was associated with CRC risk in clinical trials [66]. Leptin could also favour CRC growth in vivo and in vitro experiment as a pleiotrophic hormone being mitogenic, anti-apoptotic, pro-angiogenic, and proinflammatory in various cellular systems [67]. The relationship between circulating leptin concentrations and CRC risk have been demonstrated [68]. Following those finding, the association between obese and the risk of CRC has been assessed in several prospective cohort studies, with most studies showing a statistically significant positive association.

Meta-analysis is an important tool for revealing trends that might not be apparent in a single study. Pooling of independent but similar studies increases precision and therefore increases the confidence level of the findings [69]. The current meta-analysis had some advantages. First, the number of total cases were substantial, which significantly increased the statistical power of the analysis. Second, our quantitative assessment was based on prospective studies, which will minimize the possibility that our results were due to recall or selection bias. Third, the majority of the studies included in the meta-analysis evaluated multiple confounders including dietary pattern, physical activity, alcohol drinking, smoking, and other factors. The relationships between BMI/WC and CRC risk in each study were derived after adjustment at least for age. The pooled estimate was stable and robust after comprehensive sensitivity analyses. We evaluated the quality of the eligible studies with the Newcastle Ottawa scale, the assessment included selection of populations, comparability of cohorts and ascertainment of outcome. The studies included in our current article were considered as high quality because the total score of the studies ranged from 6–9. Finally, the large number of studies describe the detailed data of subgroup analyses, permitted us to better understand the effect of obese on various subgroups.

In spite of these advantages, some limitations of the present meta-analysis should be acknowledged. First, The variations in the BMI and WC categories between studies is a source of heterogeneity, and this may possibly lead to less accurate estimates of risk. Second, the current meta-analysis is unable to solve problems with confounding factors that could be inherent in the included studies. Inadequate participants of the confounders might bias the results in either direction toward exaggeration or underestimation of risk estimates. Although most studies adjusted for other known risk factors for CRC, unknown confounders cannot be excluded as a potential explanation for the observed findings. Third, significant heterogeneity was observed across studies, which would throw some doubt on the reliability of the summary RR estimates. This significant heterogeneity might exist in terms of study design, demographics of participants, ascertainment of anthropometry, duration of follow-ups, and confounders. We are unable to account for these differences, despite the use of appropriate meta-analytic techniques with random-effect models. Moreover, by conducting stratified analysis, we found that the risk estimates of BMI/WC and the risk of CRC were robust and stable across various study characteristics. Finally, potential publication bias is impossible to be completely excluded because small studies with null results tend not to be published.

In summary, the results from this meta-analysis of prospective studies demonstrate that BMI and WC levels are both positively associated with risk of CRC. This positive association also exists in both men and women, different geographic region, and different anatomical site. However, available data are still sparse, and in-depth analyses of the assessed associations in the context of additional longitudinal studies are highly desirable to enable more-precise estimates and a better understanding of the role of obesity in CRC carcinogenesis. The findings from these observational studies need to be confirmed in large randomized clinical trials in the future.

## Supporting Information

Checklist S1 **PRISMA 2009 Checklist.**
(DOC)

Diagram S1 **PRISMA 2009 Flow Diagram.**
(DOC)
