# Supplementary material for: Obesity and Risk of Colorectal Cancer: A Systematic Review of Prospective Studies
Source: PLoS One. 2013 Jan 17;8(1):e53916. doi: 10.1371/journal.pone.0053916 (PMC3547959; doi:10.1371/journal.pone.0053916)
Supplement: Diagram S1 — PRISMA 2009 Flow Diagram. (DOC) [file pone.0053916.s002.doc]

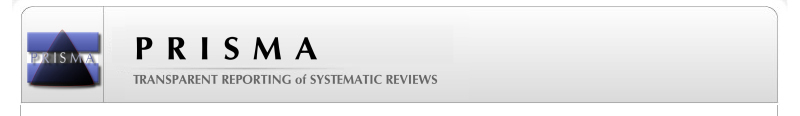
**PRISMA 2009 Flow Diagram**

**Screening**

**Included**

**Eligibility**

**Identification**

Records identified through database searching
(n = 5916)

Additional records identified through other sources
(n = 0 )

Records after duplicates removed
(n = 3385 )

Records screened
(n = 341 )

Records excluded
(n = 298 )

Full-text articles assessed for eligibility
(n = 41 )

Full-text articles excluded, with reasons
(n = 0 )

Studies included in qualitative synthesis
(n = 41 )

Studies included in quantitative synthesis (meta-analysis)
(n = 41 )
